# Minimally invasive plate osteosynthesis of the humeral shaft with regard to adjacent anatomical characteristics

**DOI:** 10.1038/s41598-021-04041-w

**Published:** 2022-01-07

**Authors:** Gloria Maria Hohenberger, Georg Lipnik, Angelika Maria Schwarz, Peter Grechenig, Magdalena Holter, Andreas Heinrich Weiglein

**Affiliations:** 1 Department of Trauma Surgery, State Hospital Feldbach-Fürstenfeld, Ottokar-Kernstock-Straße 18, 8330 Feldbach, Austria; 2 Division of Macroscopic and Clinical Anatomy, Gottfried Schatz Research Centre, Medical University of Graz, Harrachgasse 21, 8010 Graz, Austria; 3 AUVA-Trauma Hospital (UKH) Styria|Graz, Teaching Hospital of the Medical University of Graz, Göstingerstraße 24, 8020 Graz, Austria; 4 Department of Orthopaedics and Traumatology, Paracelsus Medical University, Müllner Hauptstraße 48, 5020 Salzburg, Austria; 5 Institute for Medical Informatics, Statistics and Documentation, Medical University of Graz, Auenbruggerplatz 2, 8036 Graz, Austria

**Keywords:** Musculoskeletal system, Bone

## Abstract

The study goal was to evaluate the distances from the radial (RN), the musculocutaneous (MN) and axillary nerves (AN) and the medial neurovascular bundle of the upper arm to a minimally invasive applied plate and to define its relation to the RN during different degrees of malrotation during MIPO. The sample involved ten upper extremities. Application of a PHILOS plate was performed through a Delta-split. Intervals between the AN, MN, RN and the medial vascular bundle were defined at various positions. The humeral shaft was artificially fractured at a height of about the mean of the plate. The distal fragment was brought into 15° and 30° internal (IR) as well as external rotation (ER) and here, the plate’s relation to the RN was evaluated. The medial neurovascular bundle intersected the plate at its distal part in two specimens. Regarding the distances from the RN to the plate during different rotation positions the distances became significantly longer during ER, respectively shorter during IR. The medial neurovascular bundle and the RN were identified as the main structures at risk. Care must be taken during distal screw placement and malrotation exceeding 15° must be avoided during MIPO.

## Introduction

Fractures of the shaft of the humerus represent common injuries, involving 1% to 3% of all fractures, respectively 20% of all fractures of the humerus^1–3^. Most of these may be treated conservatively. However, the non-operative approach includes the risk of secondary loss of reduction and non-union rates ranging from 2% up to 23% have been reported^4^. Regarding surgical treatment, plate osteosynthesis and antegrade intramedullary nailing are the most common options. Nevertheless, controversy about the ideal interventional technique still exists. Although open reduction and internal fixation (ORIF) enables anatomical reduction, it includes extensive soft tissue dissection^4–6^ and despite nerve protection radial nerve (RN) palsies have been described in 5.1–17.6%^6^. Antegrade intramedullary nailing, although representing a percutaneous method^4,7^, may lead to rotator cuff lesions with postoperative shoulder dysfunction^8^, whereas retrograde nailing may be followed by pain around the elbow region. Additionally, minimally invasive plate osteosynthesis (MIPO) has become a popular alternative to these common therapy options. This technique has led to satisfactory outcomes in several clinical trials^2,6,7,9,10^. It enables minor soft tissue damage in comparison to open methods, earlier postoperative functional treatment^2^ and biological fracture healing^9^. Further, fewer infections and cases of RN palsy have been reported^4^. Nevertheless, Wang and colleagues^3^ observed an increased postoperative malrotation of the humeral shaft, exceeding 20°, during MIPO in comparison to an ORIF control group.

**Figure 1:**
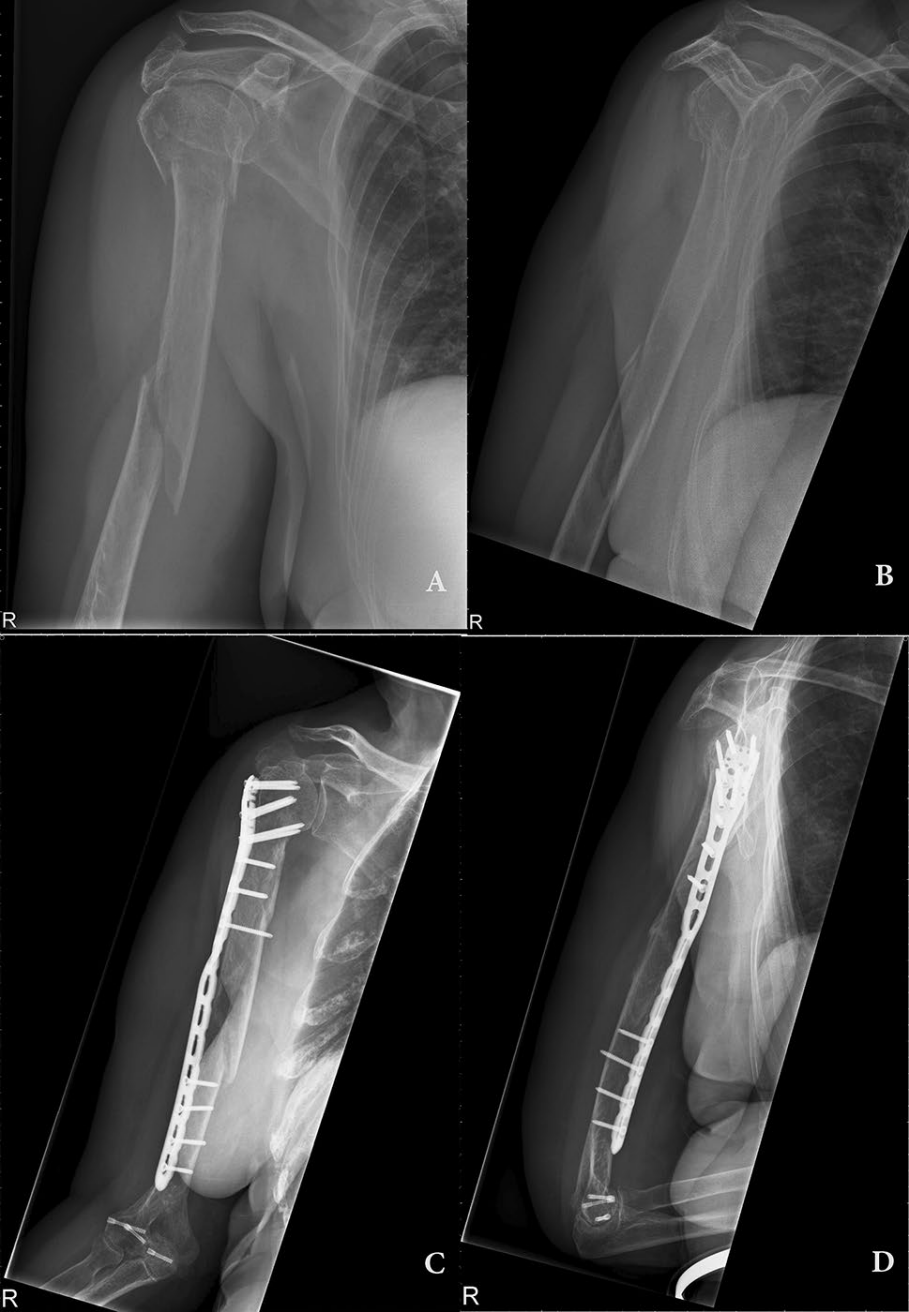
Clinical example of a pre-contoured helical plate in a female 79 years old patient. (**A**) and (**B**) preoperative X-ray of a proximal humeral fracture with meta-/diaphyseal extension. (**C**,**D**) Six month postoperative X-ray with a helical PHILOS plating system.

A helical plate has the potential to avoid an iatrogenic deltoid muscle insertion lesion as well as muscular nerve palsy due to its design. An example from helical MIPO clinical setting is pictured in Fig. 1. The current literature lacks detailed information regarding the proximity of neurovascular structures to the osteosynthesis material after its minimally invasive application in neutral and different rotation positions. Therefore, the purpose of our study was to evaluate the distances from the RN, the musculocutaneous (MN) and axillary nerves (AN) and the medial neurovascular bundle of the upper arm to the applied plate. Furthermore, we aimed to define its relation to the RN during humeral shaft fractures while simulating different degrees of malrotation after MIPO.

## Materials and methods

### Study sample

The study sample involved ten unpaired upper extremities from ten human adult cadavers embalmed using Thiel’s solution^11^. All investigated corpses were donated to the Division of Macroscopic and Clinical Anatomy of the Medical University of Graz under the approval of the Anatomical Donation Programme and accordingly to the Austrian curial law. Since these bodies are dedicated to research and scientific teaching, the donors accepted participation in research during their lifetime. All experimental protocols and methods were carried out in accordance with relevant guidelines and regulations. The sample consisted of three limbs from female and seven upper extremities from male donors. Their age ranged from 49 to 92 years with a median of 82. Via inspection of the skin and soft tissues as well as through radiographic control with the C-arm, extremities with former fractures, surgical interventions or malformations in the region of interest were excluded.

### Plate application

Primary, the humeral length (HL), which was defined as the distance between the lateral humeral epicondyle (LE) and the tip of the greater tubercle, was evaluated. For implantation, we used a 12-hole PHILOS plate (Synthes GmbH, Oberdorf, Switzerland) which’s middle third had been twisted in a ventral direction in an angle of about 70°–90° over a distance of two plate holes. Access to the proximal part of the humerus was gained through an about six centimetres long skin incision and a Delta-split approach in typical manner. Here, the AN was depicted and countermined with a raspatory. Care was taken not to manipulate the nerve’s original course. Next, the plate was inserted and advanced epiperiosteally to the deltoid tuberosity and the ventral humeral surface. The PHILOS plate was applied to the bone through K-wires about 4 mm posterior to the bicipital grove. The approach to the distal portion of the plate was gained through an about 5 cm skin incision at the distal antero-lateral part of the upper arm which was conducted directly superficial to the palpable plate. Here, after splitting of the fascia, the biceps was retracted in a medial direction following depiction of the MN. The brachial muscle was split at its median part directly superficial to the plate. Here, fixation was conducted through one conventional screw.

### Measurement pattern

All measurements were conducted in millimetres and by use of a digital calliper rule. The distance between the proximal margin of the PHILOS plate and the AN as well as the interval between the tip of the greater tubercle and the AN were measured. Additionally, we observed the location of the nerve with regard to the plate’s screw holes.

After plate application, the relationship between the MN and the plate was evaluated. Therefore, the distal skin incision was advanced in a proximal direction. Primary, the ventral crossing point of the plate and the nerve was evaluated and its height with regard to the LE and the distal tip of the plate was measured. Further the distance between the medial margin of the plate and the exit point of the MN from the coracobrachialis was evaluated.

**Figure 2:**
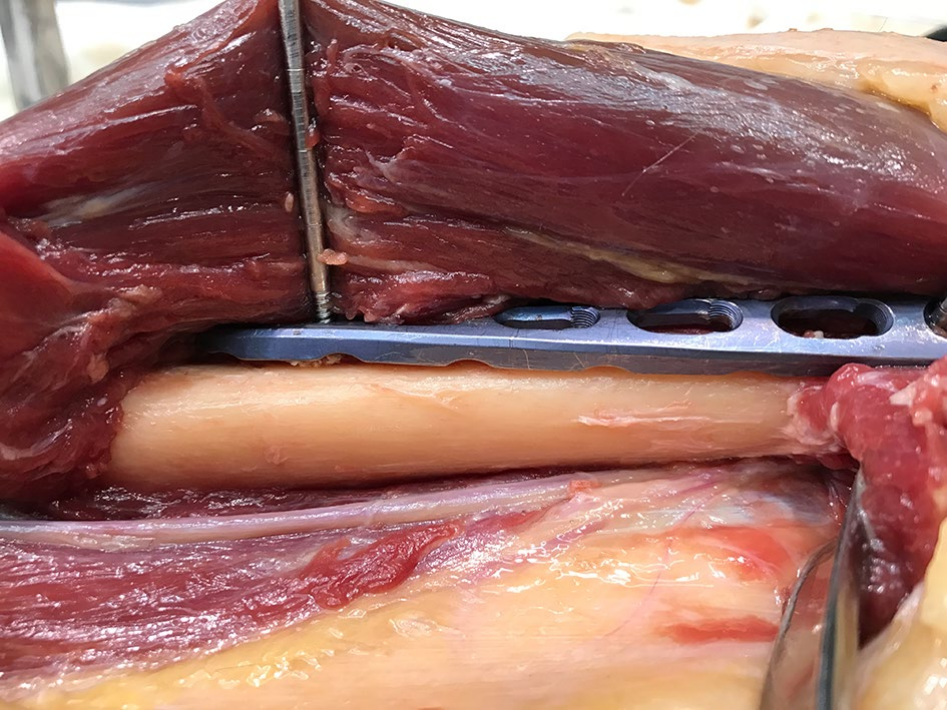
Applicated plate with adjacent RN.

**Figure 3:**
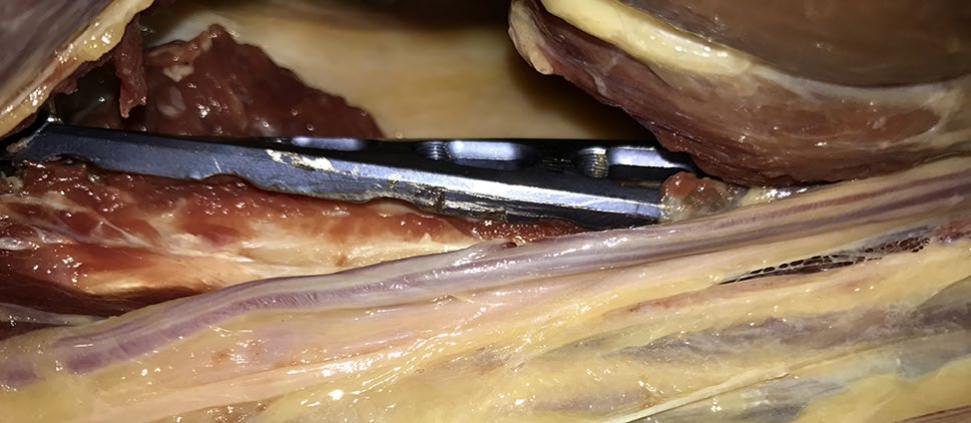
Medial vascular bundle of the upper arm in relation to the minimally invasive inserted plate.

**Figure 4:**
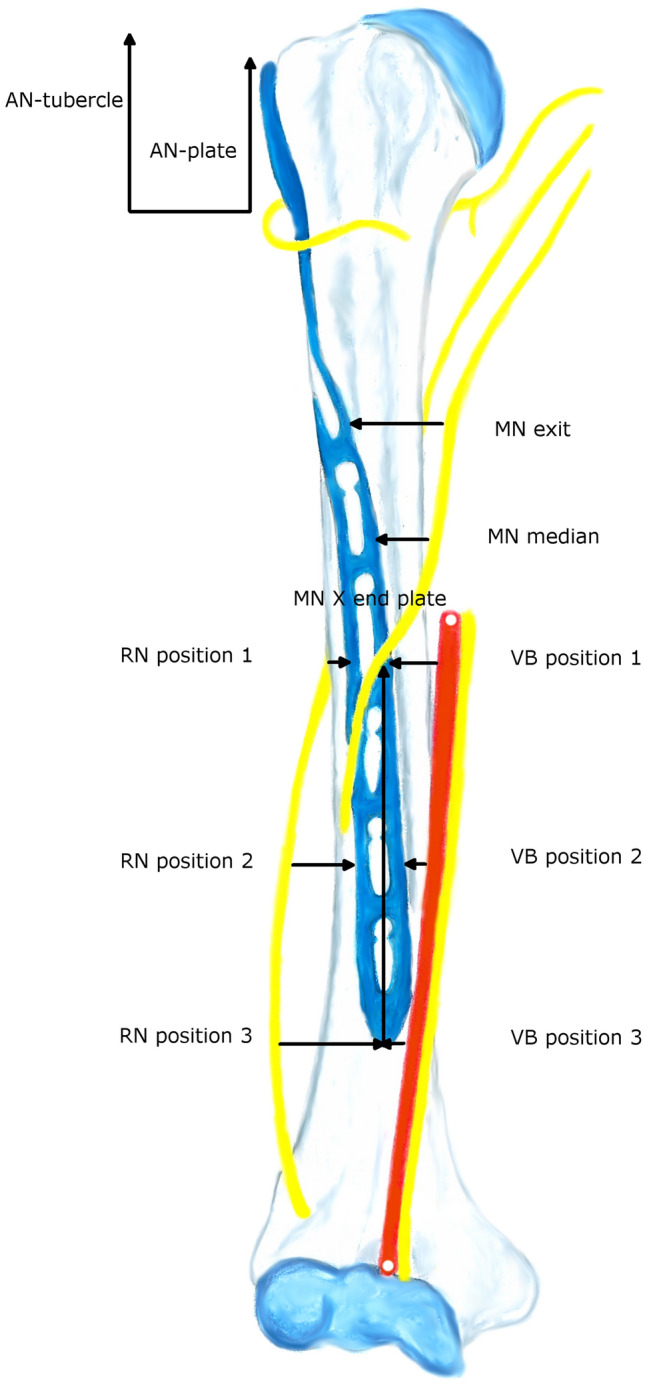
Measurement pattern. *AN-plate* AN to proximal margin of the plate, *AN-tubercle* AN to tip of greater tubercle, *VB* medial vascular bundle for respective position, *MN exit* exit of MN from coracobrachialis, *MN X end plate* intersection point of the MN and the plate measured from the distal margin of the plate, *MN median* measurement point at the middle of the exit and intersection point.

For defining the distances between the plate and the RN, respectively the medial neurovascular bundle three distances were defined: (1) The interval between the medial/lateral border of the plate and the structures at the height of the RN’s exit from the lateral brachial septum, (2) the distance between the plate and the structures at the median point between 1 and 3 and (3) the interval between the medial/lateral edge of the plate and the characteristics at the height of the distal tip of the plate (Figs. 2, 3). For measurement of the RN, the brachialis muscle was split at its median part and the two halves were retracted. For a schematic depiction see Fig. 4.

### Rotation tests of the RN

After collection of the measurements, the humeral shaft was artificially fractured at a height of about the mean of the plate by use of a circular saw. The distal fragment was brought into 15° internal (IR) and external rotation (ER) as well as 30° IR and ER. In each rotation position, the plate was re-fixed to the distal humeral surface and the distances to the RN were measured at the (above described) positions 1–3.

### Statistical analysis

Statistical analysis was conducted using SPSS statistical software (version 24.0; IBM Corp, Armonk, NY, USA). Regarding descriptive statistics, continuous variables are presented as mean, standard deviation (SD), minimum and maximum, whereas categorical data are described as frequencies and percentages. All continuous data were normally distributed. Further, a repeated measurement ANOVA including degrees of distal fracture fragment rotation and radial nerve position as within subject factors was conducted. For comparison of the three different distances between the RN and the plate at different degrees of rotation, t-tests for paired samples were used. Bonferroni adjustment for multiple testing was performed (alpha = 0.05/12 = 0.004).

## Results

### AN evaluation

The mean distance from the AN to the proximal margin of the plate proved to be 38.9 mm (SD 3.5; range 33.2–44.9) and the interval between the nerve and the tip of the greater tubercle was 48.2 mm (SD 4.9; range 40.5–55.2), see Table 1. Regarding the relation between the AN and the screw holes, the nerve’s most common location was at the proximal of the so-called calcar screws (5 cases/50%).

**Table 1:** Measurements in neutral position (*in mm*).

	AN-plate	AN-tubercle	VB position 1	VB position 2	VB position 3	RN position 1
Mean	38.9	48.2	16.6	15.2	13.6	8.2
SD	3.5	4.9	9.4	12.1	13.6	3.5
Median	40.0	47.5	17.1	13.7	12.0	8.0
Min	33.2	40.5	1.0	− 2.0	− 5.0	4.0
Max	44.9	55.2	33.0	36.0	35.0	15.0

	RN position 2	RN position 3	MN exit	MN X end plate	MN X LE	MN median
Mean	6.4	8.1	14.9	52.5	82.9	13.7
SD	1.5	2.3	4.5	16.0	19.4	9.9
Median	6.5	8.0	16.0	53.8	77.5	10.4
Min	4.0	5.0	4.2	28.0	59.0	0.0
Max	9.0	12.0	20.6	80.0	120.0	29.0

*AN-plate* AN to proximal margin of the plate, *AN-tubercle* AN to tip of greater tubercle, *VB* medial vascular bundle for respective position, *MN exit* exit of MN from coracobrachialis, *MN X end plate* intersection point of the MN and the plate measured from the distal margin of the plate, *MN X LE* intersection point of the MN and the plate measured from the LE, *MN median* measurement point at the middle of the exit and intersection points, *SD* standard deviation, *min* minimum, *max* maximum.

### MN and neurovascular bundle measurements

The mean humeral length was 30.8 cm (SD 3.5) with a range from 23.4 up to 34.6 cm. The distance between the medial neurovascular bundle to the plate was on average 16.6 mm (SD 9.4; range 1–33) at position 1 and 15.2 mm (SD 12.1; range – 2 to 36) at position 2. Regarding position 2, the bundle lay on the plate in one case (with 2 mm from its medial border to the medial margin of the plate; value: − 2 mm). At position 3, the mean interval was 13.6 mm (SD 13.6) with a range from – 5 to 35 mm. Here, the bundle intersected the plate in two specimens.

The distance between the MN and the plate was on average 14.9 mm (SD 4.5; range 4.2–20.6) at its exit point from the coracobrachialis. Regarding its intersection point with the plate, this was at a height of 52.5 mm (SD 16.0; range 28–80) measured from the distal border of the plate, and 82.9 mm (SD 19.4; range 59–120 mm) with regard to the LE. Further, the plate-MN distance was 13.7 mm (SD 9.9 mm; range 0–29) at the median of the two measurement points. Details see Table 1.

### RN measurements and rotation tests

**Figure 5:**
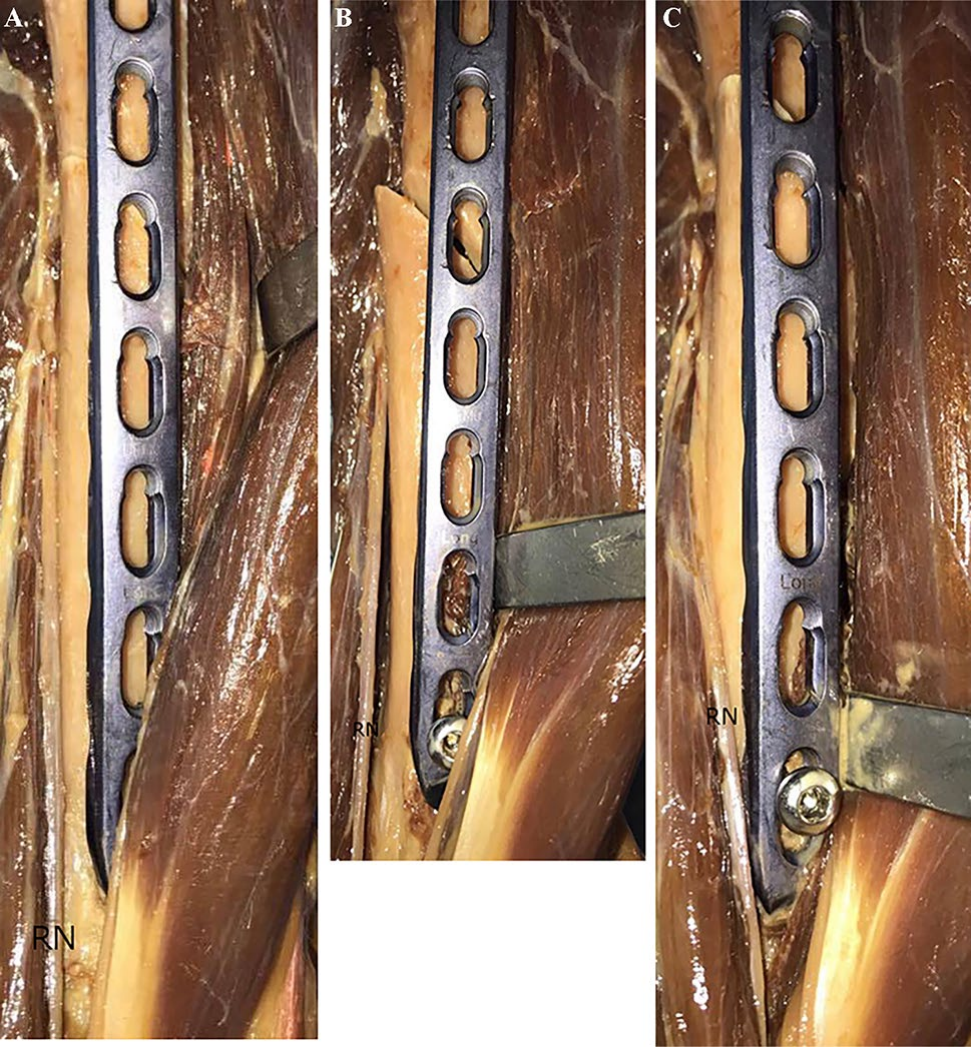
Rotation tests of the RN. (**a**) Neutral position; (**b**) external rotation; (**c**) internal rotation.

Repeated measurement ANOVA revealed statistically significant differences regarding the distances from the RN to the plate during the neutral position against all of the different rotation positions of the distal fracture fragment (p =  < 0.001). Thereof, distances became significantly longer during ER, respectively shorter during IR (see Fig. 5).

With respect to the three different measurement positions, t-test for paired samples showed significantly shorter distances during 15° and 30° IR in comparison to the neutral position for position 1 (p = 0.001). For position 2, IR and ER of 30° revealed significant differences (internal: p =  < 0.001; external: p = 0.001). Regarding position 3, all of the rotation positions showed statistically significant differences in comparison to the neutral position (p =  < 0.001). For details see Table 2.

**Table 2:** Descriptive analysis and outcomes of t-tests for RN testings (*in mm*).

Position	Mean	SD	Median	Min	Max	Paired differences		SE mean	df	p-value
						Mean	SD			
**1**										
ER 15°	9.8	2.3	9.0	7	14	− 1.6	3.4	1.1	9	.172
ER 30°	11.6	4.5	10.5	3	19	− 3.4	4.8	1.5	9	.051
IR 15°	3.1	1.8	2.5	1	6	5.1	3.5	1.1	9	.001
IR 30°	2.1	1.5	2.0	0	5	6.1	3.8	1.2	9	.001
**2**										
ER 15°	12.7	5.4	11.0	6	22	− 6.3	5.6	1.7	9	.006
ER 30°	15.1	5.6	13.5	9	24	− 8.7	5.5	1.7	9	.001
IR 15°	5.0	3.4	4.0	1	11	1.4	3.7	1.2	9	.268
IR 30°	2.0	2.2	2.5	− 2	5	4.4	2.6	0.8	9	< .001
**3**										
ER 15°	11.5	2.6	12.0	8	16	− 3.4	2.0	0.6	9	< .001
ER 30°	15.1	2.9	15.0	9	20	− 6.9	2.0	0.9	9	< .001
IR 15°	5.3	1.6	5.5	2	8	2.8	1.6	0.5	9	< .001
IR 30°	0.4	2.4	0.0	− 3	4	7.7	2.9	0.9	9	< .001

*ER* external rotation, *IR* internal rotation, *SD* standard deviation, *min* minimum, *max* maximum, *SE mean* standard error of the mean, *df* degrees of freedom.

Further, the RN was located on the plate in one specimen at position 2 (two millimetres medial to the lateral border of the plate; value: − 2), respectively in two cadavers at position 3 (both three millimetres medial to the lateral edge; value: − 3).

## Discussion

MIPO by use of curved implants has recently become a more frequent technique in the treatment of humeral shaft fractures and has resulted in satisfactory clinical outcomes in several studies^2,6,7,9,10^. The proximity of single nerves to the osteosynthesis material during MIPO has been described in the current literature^12,13^. However, the intervals between the nerves and the plate at different positions have not been delineated in detail so far.

Gardner and colleagues^13^ have declared the MN to be the main structure at risk during MIPO. Therefore, they defined a safe zone for its crossing point of the anterior humeral shaft at an interval between 12.2 and 14.8 cm measured from the greater tubercle. We in contrast, found its intersection point with the plate at a height of 52.5 mm starting from the distal border of the plate, respectively 82.9 mm with regard to the LE.

In Křivohlávek et al.’s^14^ dissection study, a PHILOS plate was applied in 24 specimens through the minimally invasive anterolateral approach. Here, the AN did not suffer injuries in any of the cases. As we performed the plate insertion through a typical Delta-split approach including depiction of the nerve, evaluation of possible iatrogenic lesions was not possible. The mean distance from the AN to the tip of the greater tubercle was at a mean of 48.2 mm with a range from 40.5 up to 55.2 mm in our survey. In comparison, Křivohlávek et al.’s^14^ intervals were shorter, ranging from 37 to 44 mm. Further, our evaluated mean distance from the AN to the proximal margin of the plate proved to be 38.9 mm which was also slightly greater than in Ninck et al.’s^15^ dissections (mean of 36.1 mm). With regard to the AN in relation to the proximal screw holes, we evaluated the nerve’s most common location (50%) on the calcar screws. This was also the most frequent position in Křivohlávek et al.’s^14^ study, however, Ninck and colleagues^15^ found the AN in most of the cases more distal at region F.

Regarding the medial vascular bundle of the upper arm, we observed the location of this structure on the plate in one specimen at position 2, respectively in two cases at position 3—a fact that needs to be considered during distal screw placement.

With respect to tolerated malrotation during conservative treatment of humeral shaft fractures, this was suggested not to exceed 15°^16,17^. Further, a postoperative malrotation exceeding 20° was described to be associated with secondary shoulder arthritis^18,19^. Wang et al.^3^ observed a significant increase of postoperative malrotation after MIPO in comparison to their ORIF control group. Additionally, the concomitant rotation of the RN during IR and ER of the distal fracture fragments needs to be considered. Our conducted repeated measurement ANOVA showed a statistically significant increase of the intervals between the RN and the lateral border of the plate at the three defined positions during ER, whereas they became significantly shorter during IR. During IR of 30°, we observed the location of the RN directly on the plate in one case at position 2 and in two specimens at position 3.

In conclusion we have examined the relation of a minimally invasive inserted plate to adjacent neurovascular characteristics of the upper arm. We have identified the medial neurovascular bundle and the RN (during IR) as the main structures at risk. Therefore, care must be taken during distal screw placement and malrotation exceeding 15° needs to be avoided. To prohibit the latter, we recommend to place the upper extremity under continuous longitudinal tension and to perform regular X-ray controls during surgery.
